# Addressing harmful gender norms in sexual health services

**DOI:** 10.2471/BLT.23.291155

**Published:** 2024-10-29

**Authors:** Rosemary Morgan, Anna Kalbarczyk, Martha de la Paz, Evelyn Boy-Mena, Anna Coates

**Affiliations:** a Department of International Health, Johns Hopkins Bloomberg School of Public Health, Baltimore, United States of America.; b Department of Gender, Rights, Equity and Diversity, World Health Organization, Avenue Appia 20, 1211 Geneva 27, Switzerland.

Gender norms regarding sexuality, sexual agency and bodily autonomy dictate what behaviours, roles and attributes are considered appropriate for women and girls, men and boys and transgender individuals.^1^ These norms vary according to culture and context, and are fundamental to upholding patriarchal systems and structures of power which privilege men and boys. They are embedded within institutions and organizations and are reinforced and sustained through interpersonal interactions,^1^ including within sexual health services.

Access to appropriate sexual health services is fundamental for upholding the sexual rights of cisgender women, girls and transgender individuals, protecting their rights to fulfil and express their sexuality and enjoy sexual health.^2^ In low-resource settings, sexual health services remain largely unavailable.^3^ When they are present, cisgender women, girls and transgender individuals can face barriers due to pervasive stigma, bias and discrimination within health systems and by health workers. 

Stigma, bias and discrimination are related concepts that operate on structural, interpersonal and individual levels. Stigma arises from negative societal perceptions and manifests as stereotyping, labelling, judgment and rejection.^4^ Bias is the tendency to favour one group over another or to have prejudice against a particular group. Discrimination is defined as unfair and unjust actions towards an individual or group based on stigma and bias. Gender-related discrimination stems from the desire to maintain dominant norms in patriarchal societies, marginalizing cisgender women, girls and transgender individuals for their non-conformity to restrictive gender norms.^5^

In this article, we argue that addressing inequitable gender norms surrounding sexual health services is crucial to avoiding negative health outcomes and promoting the sexual agency and bodily autonomy of cisgender women, girls and transgender individuals. We outline ways in which gender-related stigma, bias and discrimination manifest within sexual health services, as well as potential solutions. Tackling harmful gender norms that underlie stigma, bias and discrimination can help promote the rights of cisgender women, girls and transgender individuals by upholding their sexual agency and bodily autonomy, and contribute to achieving more inclusive and equitable sexual health services for all.

## Manifestations of harmful gender norms

Health systems where traditional gender norms and roles are rewarded can reinforce inequitable gender norms, where for example cisgender women are expected to engage in sexual activity primarily to satisfy the maternal role as opposed to being autonomous sexual beings.^6^ These biases can manifest as the unavailability of sexual health services because of legal and policy barriers and/or deprioritization. For example, comprehensive sexual health services remain absent in essential health service packages in many low- and middle-income countries.^3^ When services are available, restrictive gender norms can still affect access because of biases, leading to differential treatment and judgment based on gender identity in provider-patient interactions. Cisgender women’s sexual health concerns, including pain related to sexual illnesses or reproductive health, are often dismissed as psychological, while cisgender men may receive more immediate attention for similar conditions.^1^^,^^7^ Meanwhile, transgender individuals can experience misgendering and negative attitudes when seeking gender-affirming care such as hormone therapy or genital and chest reconstruction surgery.^8^

Beyond unequal treatment, gender biases towards these populations can manifest as violence and abuse within and beyond the health-care system. Cisgender women, girls and transgender individuals experience high levels of gender-based violence, including sexual violence,^8^^,^^9^ requiring access to responsive health services for their unique needs. However, health workers have been documented perpetrating verbal, physical, sexual and economic abuse.^7^ Verbal abuse tends to occur when cisgender women transgress certain gender norms, for example when seeking sexual health services when unmarried, or seeking services for sexual abuse.^7^ Economic abuse includes demanding under-the-counter payments for services and medications, exacerbating existing financial barriers for many cisgender women and transgender individuals. Linked to economic abuse is the overmedicalization of women’s health, where providers overprescribe due to perverse financial incentives and/or stereotypes about women’s bodies.^7^ Practices like female genital mutilation, child marriage and criminalizing abortion attempt to reduce the function of cisgender women’s and girl’s bodies to childbearing and to cisgender men’s sexual pleasure, undermining their sexual agency and bodily autonomy.^10^ Blatant abusive practices towards transgender individuals, such as non-consensual disclosure of their gender identity (outing), dismissal, delayed provision of care and conversion therapy foster internalized shame, and suppress transgender identities and their gender expressions.^9^ Overmedicalization and hypervigilance are less straightforward and often overlooked means of erasure of transgender identities, which forces transgender individuals to conform to cisnormative bodies, and further marginalizes those with non-binary identities and expressions.^9^

Lack of access to sexual health services, whether due to unavailability or barriers created by restrictive gender norms, leads to harmful health practices in these populations, such as unregulated self-medication, self-surgery, use of black-market hormones and delayed or foregone care. These practices can result in severe negative health impacts, including avoidable suffering and deaths. Experiences of stigma, bias and discrimination when accessing and using sexual health services can affect an individual’s willingness to seek care.^6^ In many contexts, cisgender women and girls, particularly adolescent girls and unmarried women and girls, avoid seeking treatment for sexually transmitted infections due to fear of stigma and negative treatment by health providers.^6^ Transgender individuals may delay or avoid care due to past experiences of discrimination.^1^ These issues can be compounded by intersectional biases such as when racial and ethnic minority groups experience exacerbated stigmatization.^7^ Cisgender women, girls and transgender individuals may also resort to seeking alternative forms of care outside the formal health system, such as traditional providers or self-care. Although these options can promote self-determination and empowerment when supported and part of an informed choice,^11^ they often result from inaccessibility and can have adverse effects. The broader ramifications of these experiences affect sexual agency and bodily autonomy for both cisgender women and girls, as well as transgender individuals. By denying an individual’s sexual agency and bodily autonomy, health systems and service providers help maintain dominant power asymmetries by perpetuating harmful gender norms.

## Employment of harmful gender norms

Experiences of stigma, bias and discrimination vary according to identity with a fundamental difference in their intent. Gender norms for cisgender women and girls are employed to exert control as they are often seen as reproductive vessels – as opposed to autonomous individuals with separate sexual desires, needs and rights.^5^ Meanwhile, gender norms for transgender individuals are employed towards erasure as they are perceived to undermine dominant constructs related to the ways in which sex characteristics correspond to gender identity and gender expression, and thus the relationship between reproduction and sexuality.^5^ The common thread of these forms of stigma, bias and discrimination is the censure of those who do not embody societal gender norms regarding the relationship between sexuality and reproduction.

In this article, we have described interpersonal manifestations of discrimination; however, noting that these are rooted in wider structural issues stemming from societal attitudes and inequalities is important. Interpersonal interactions are merely the way in which these are enacted, experienced and perpetuated within systems. Providers themselves are not exempt from prejudice and discrimination;^12^ many health and care providers, the majority of whom are women, face discrimination in the workplace. For example, a study showed that female health workers are underpaid, undervalued, under-promoted and forced to work in unsafe environments.^12^ This situation can affect how providers interact with patients, feeding into the many discriminatory practices listed throughout this paper.

## Dismantling harmful gender norms 

Sexual agency and bodily autonomy are important gender equality outcomes for cisgender women, girls and transgender individuals. Sexual health services should seek to dismantle harmful gender norms by promoting and supporting sexual agency and bodily autonomy for these groups. Within health systems, cisgender women, girls and transgender individuals need to be seen as persons with sexual desires, needs and rights beyond their reproductive function.^5^ Capacity-building for health workers can increase awareness of discriminatory practices stemming from gender norms and improve their competencies. Participatory approaches, that is, approaches designed with or by a stigmatized group, have been documented to improve patient-provider relationships.^6^^,^^7^ Specialized clinics or services for women’s health and transgender health can be established to improve the availability of sexual health services for these populations, especially in low-resource settings. Integrating sexual health services into existing services can further improve availability of care.^7^^–^^9^ Policies aimed at reducing stigma, bias and discrimination should be prioritized to mandate safe and inclusive environments.

To support policy and programme recalibration, efforts led by research institutions, health organizations, community-based nongovernmental organizations and government agencies to broaden research in various settings providing sexual health care at different levels must be pursued. Existing literature focuses on measuring and addressing individual and interpersonal manifestations of stigma and discrimination, with less attention given to policies.^6^ Furthermore, assessments of biases, barriers and gaps in service delivery across a variety of sexual health settings are needed to better understand and intervene in these issues in different contexts.^6^ Such concerted action, aligned with global health goals and targets, will support health systems and the pledges of policy-makers, government authorities and health ministries to improve sexual health outcomes for all as part of their commitment to universal health coverage. 
